# Infection control knowledge, beliefs and behaviours amongst cystic fibrosis patients with epidemic *Pseudomonas aeruginosa*

**DOI:** 10.1186/s12890-015-0116-x

**Published:** 2015-11-05

**Authors:** R. Somayaji, B. Waddell, M. L. Workentine, M. G. Surette, N. P. Brager, H. R. Rabin, M. D. Parkins

**Affiliations:** Department of Medicine, The University of Calgary, Calgary, Canada; Department of Microbiology, Immunology and Infectious Diseases, The University of Calgary, 3330 Hospital Drive, NW, Calgary, AB Canada; Department of Veterinary Medicine, The University of Calgary, Calgary, Canada; Department of Psychiatry, The University of Calgary, Calgary, Canada; Department of Medicine, McMaster University, Hamilton, ON Canada; Department of Biochemistry, McMaster University, Hamilton, ON Canada; Farncombe Family Digestive Health Research Institute, McMaster University, Hamilton, ON Canada

**Keywords:** Liverpool epidemic strain, Prairie epidemic strain, Infection transmission, Transmissible strains

## Abstract

**Background:**

Epidemic *P. aeruginosa* (ePA) infections are common in cystic fibrosis (CF) and have been associated with accelerated clinical decline. Factors associated with ePA are unclear, and evidence based infection control interventions are lacking.

**Methods:**

We prospectively collect all bacterial pathogens from adult CF patients. We performed PA strain typing on retrospectively collected enrolment samples and recent isolates to identify patients infected with ePA. All patients attending our clinic were approached to complete a survey on infection control knowledge, beliefs and exposures. We analyzed responses of those with ePA relative to the entire cohort without ePA as well as those infected with unique strains of *P. aeruginosa* to assess for risk factors for ePA and differences in infection control knowledge, beliefs or behaviours.

**Results:**

Of 144 participants, 30 patients had ePA (two Liverpool epidemic strain, 28 Prairie epidemic strain), 83 % of which had established infection prior to transition to the adult clinic. Risk of concomitant infecting pathogens was no different between groups although, *Staphylococcus aureus* and non-tuberculous mycobacteria were less common in those with ePA. Patients with ePA were more likely to have attended CF-camp and have a history of CF fundraising. Patients with ePA did not differ with respect to beliefs regarding pathogens or transmission risk, except they believed indirect contact posed little risk. Furthermore, patients with ePA were more likely to continue to associate with others with CF despite extensive counselling. Use of peer-peer online networking was minimal in both groups.

**Conclusion:**

Infections with ePA are closely linked to past exposures, now routinely discouraged. As socialization is the greatest risk factor for ePA, infection control strategies for ePA must focus on discouraging face-to-face interactions amongst CF patients. As peer support remains a desire amongst patients, investment in technologies and strategies that enable indirect communication and support are required.

## Background

Cheng et al. are largely recognized as identifying the first epidemic *P. aeruginosa* (ePA) infecting individuals with CF [1]. These researchers identified disproportionally high rates of ceftazidime resistance amongst *P. aeruginosa* isolated from their pediatric population and subsequently genotyped all isolates using molecular methodology identifying 85 % of patients harbored the same strain, the Liverpool Epidemic Strain (LES). LES has since been identified to be widespread in clinics across the United Kingdom, and in Eastern Canada [2–5]. Furthermore, multiple other ePA strains have since been described in Australia, Europe and North America [2, 3, 6–9].

Patients chronically infected with many of the ePA strains are associated with a worsened clinical course relative to those infected with unique, non-clonal strains. In particular, LES has been associated with increased rates of lung function decline, exacerbation frequency and progression to end stage lung disease [2, 10, 11]. We have recently described another ePA, termed the Prairie Epidemic Strain (PES), amongst patients attending the Calgary Adult Cystic Fibrosis Clinic. PES is unique to CF, and was not found causing infection in comparator populations of adults with non-CF bronchiectasis, or community-acquired bacteremia nor during extensive sampling of natural environmental reservoirs or the local hospital sampling [7, 12]. This strain was evident at first encounter amongst multiple patients transferring to our adult clinic from other Western Canadian CF clinics suggesting PES is prevalent across the Prairies [7]. PES was observed to exist in even the earliest samples collected in 1980 (unpublished observations), and has been identified in sequential cohorts of young adults transitioning into our clinic. Most importantly, infection with PES occurred almost universally prior to adulthood, and very few cases of super-infection were documented in an adult cohort despite prolonged follow-up [7]. Like LES, PES has been associated with a worse baseline lung function and nutritional status, increased rates of lung function decline and progression to end stage lung disease, and are more likely to be resistant to antibiotics [7, 13].

Risk factors for ePA are poorly understood. One of the major limitations is lack of historical context to determine when infections have occurred as most ePA related studies are prospective and of short duration. As we were previously able to establish infections occur generally prior to adulthood we sought to determine what behaviours and exposures associated with ePA infection. Few studies have been conducted on patient beliefs and behaviours with respect to infection control [14]. To date no published works have sought to determine if patients with transmissible pathogens in CF have different behaviours and beliefs that may explain their infection status and contribute to risk for spread. Accordingly we sought to prospectively assess attitudes, behaviours and beliefs amongst our clinic cohort regarding infection control to identify potential factors that may lead to lapses in infection control.

## Methods

### Patient population

The Adult Cystic Fibrosis Clinic at the Foothills Medical Center, established in 1978, follows and provides all primary and specialty CF care to those with CF residing in Southern Alberta, Canada. Upon enrolment in our clinic, all patients provide prospective consent for the collection, storage and study of sputum and sputum-derived organisms (CHREB E-23087). This established prospectively collected biobank includes all morphologically distinct bacterial isolates from each and every encounter dating back to 1978. Accordingly we have been able to identify infecting pathogens and perform genotyping on *P. aeruginosa* upon enrolment in the clinic and from the most recently available clinical samples [7].

### Infection control strategy

Prior to 2010 the focus on infection control in the clinic merely involved vigorous adherence to hand and cough hygiene, as well as maintaining a 1 m “protective bubble” between patients. In 2010, these principles were expanded to include mandatory segregation of all patients to private exam rooms (including spirometry) with no possibility for co-mingling, an expansion of the “protective bubble” to 2 m in size, and contact isolation for individuals who had cultured any methicillin resistant *Staphylococcus aureus* (MRSA) in the prior 2 years. We perform bacterial strain typing by pulse-field gel electrophoresis (PFGE), supplemented with multi-locus sequence typing (MLST) and/or whole genome sequencing (WGS) for any suspected transmission of *P. aeruginosa* or other respiratory pathogens but have not observed any transmission events during five years of surveillance [15].

### Definitions

Chronic infection with *P. aeruginosa* was defined as per the Leeds criteria [16]. Infection with another airways pathogen was defined as at least one positive culture in the year prior to assessment, although patients on chronically suppressive *M. abscessus* therapy that no longer grew this organism were considered positive for the length of their treatment.

### Strain typing

Bacterial strain typing was performed using pulsed-field gel electrophoresis following protocols established by our group [7]. For each time point all morphotypes of *P. aeruginosa* (defined as morphologically distinct populations on MacConkey agar) were assessed. For those patients who were recipients of life saving bilateral lung transplantation, we used sputum culture samples collected immediately prior to their transplant to define their airway infection status (as other groups have established concordance between pre- and post transplant infecting *P. aeruginosa* which continue to infect the sinuses and periodically the graft) [17]. Strains deemed potentially ePA were those that existed in greater than three individuals from different kindreds, or previously recognized ePA strains. In situations where PFGE determination was questioned, MLST/WGS was used to confirm results.

### Survey

All patients attending the clinic were approached to complete a survey pertaining to infection control. In particular, past behaviours and risks for acquisition of infection were queried targeting childhood and early adulthood exposure as establishment of ePA infection have been demonstrated to have occurred earlier than age 20 within this cohort [7]. We sought to establish current knowledge and beliefs regarding infection control with respect to: individual pathogens; situations, and interventions in those with ePA relative to those with UNI (chronic stable unique *P. aeruginosa*) and ENT (the entire cohort who did not have ePA). This survey was completed using a 7-point Likert scale where the following values were used: 1. No risk, 2. Very low risk, 3. Low risk, 4. Neutral, 5. Mild risk, 6. Moderate risk, 7. Extreme risk [18, 19]. Finally, current behaviours and preferences were assessed using a 7-point Likert scale; 1. Strongly disagree, 2. Disagree, 3. Neutral, 4. Neither agree or disagree, 5. Somewhat agree, 6. Agree, 7. Strongly Agree. A complete reference to the survey is available in the appended supplemental material section. While patients were aware of their own lower airways pathogens in principle, they were not aware of whether their particular *P. aeruginosa* strains were ePA as typing occurred concurrent to the survey. Informed written consent was obtained from each participant. Ethical approval of this study was granted by the Conjoint Health Region Ethics Board (E-24123).

### Statistics

Statistical analysis was performed using Stata version 11.0 (Stata- Corp, TX, USA). Asymmetrically distributed variables were reported as medians with interquartile range (IQR) and compared using the Wilcoxon rank-sum test for pairs or the Kruskal-Wallis test for multiples. Differences in proportions among categorical data were assessed using Fisher’s exact test for pair-wise comparisons and the Chi-square test for multiple groups and reported as odds ratio (OR) with confidence intervals (CI). Significance was based on α < 0.05, and all hypothesis tests were 2-sided.

## Results

### Patient demographics

One hundred and forty-four of 169 active patients (85 %) within the clinic cohort participated in the study. Demographic and infection factors were not different between participants and those few non-participants. Median age of the cohort was 30.2 years (IQR 24.9–40.2) and 79 (55 %) were female. 25 patients (17.3 %) were recipients of saving lung transplantation. Sixty-nine percent of the cohort (99/144) had chronic *P. aeruginosa* infection as defined by the Leeds criteria. All but two patients with chronic *P. aeruginosa* underwent bacterial strain typing (98 %). Those two patients were unable to be categorized for the following reasons; one did not produce sputum (PA status determined by cough swab – but these isolates are not available for typing) and one patient transferred to our clinic following transplant and thusly pre-transplant samples were unavailable for assessment. Twenty–one percent (30/142) individuals in the cohort were chronically infected with ePA and 48 % (68/142) were infected with UNI. Of the ePA, 28 patients were infected with PES and two with LES. Patient demographic factors did not significantly differ amongst those with ePA infection relative to those with UNI or ENT (Table 1) with the exception of pancreatic insufficiency ePA 29/30 (97 %) vs non-ePA [93/112 (83 %), RR 1.16, CI 1.04–1.29. Patients with ePA were no more likely to have concurrent infection with other pathogens (Table 1). In fact, patients with ePA were less likely to have concurrent chronic *S. aureus* infection or non-tuberculous mycobacteria (NTM).

**Table 1:** Patient Demographics as a function of chronic infection with epidemic *P. aeruginosa* strains

Category	Epidemic *P. aeruginosa* (*n* = 30)	Cohort with unique chronic *P. aeruginosa* infection		Entire CF cohort without epidemic *P. aeruginosa*	
		UNI (*n* = 67)	P Value	ENT (*n* = 112)	P Value
Age	32.81 (IQR 29.5–39.3)	29.61 (IQR 24.9–41.9)	0.13	29.5 (IQR 23.7–42.5)	0.10
Gender (Female)	17 (56.7 %)	40 (60 %)	0.48	60 (53.4 %)	0.84
Pancreatic Sufficient	1 (3.3 %)	6 (9 %)	0.3	19 (17 %)	0.07
F508 del Homozygous	18 (60 %)	37 (56 %)	0.44	56 (50 %)	0.42
≥1 F508del allele	27 (90 %)	57 (66 %)	0.5	93 (83 %)	0.56
Status post lung transplant	12 (40 %)	9 (13.4 %)	0.005	12 (10.7 %)	<0.001
Age at transplant	29 (IQR 24.5–31.2)	33.27 (IQR 23.4–35.7)	0.53	33.9 (IQR 23.3–38.3)	0.41
Chronic Infection Status					
*P. aeruginosa*	30 (100 %)	67 (100 %)	N/A	67 (59.8 %)	<0.001
*S. aureus* (MSSA)	5 (20 %)	29 (43.7)	0.009	61 (54.5 %)	<0.001
MSSA as sole pathogen	0	0	N/A	21 (18.8 %)	0.007
*S. aureus* (MRSA)	1 (3 %)	3 (5 %)	0.63	8 (7 %)	0.68
*S. maltophilia*	0	0	N/A	2 (2 %)	1
Non-tuberculous mycobacteria	0	6 (9 %)	0.1	17 (15.1 %)	0.02
*M. abscessus* complex^a^	0	2 (3 %)	0.48	10 (9 %)	0.12
*M. avium* complex	0	4 (6 %)	0.22	7 (6 %)	0.34
*A. xylosoxidans*	0	0	N/A	2 (2 %)	1
*B. cepacia* complex (Bcc)^b^	1 (3 %)	2 (3 %)	0.67	4 (3.5 %)	1
*Aspergillus fumigatus*	2 (6.6 %)	2 (3 %)	0.36	5 (4.5 %)	0.64
No chronic pathogens	0	0	N/A	6 (5.3 %)	0.34

^*a*^ *M. abscessus* complex = 8 *M. abscessus abscessus*, 2 *M. abscessus bolletti*

^b^Bcc = *B. multivorans* 1, *B. cenocepacia* 3

Of patients with ePA, 25/30 acquired infection prior to transition to the adult CF clinic. In those five with ePA super-infections as an adult, these infections were acquired within the first 2 years of transfer and none occurred in the last decade outside of one siblingship where the older of the pair had established infection prior to transfer to the adult clinic (data not shown) [7]. We also assessed how many prior CF clinics patients had attended as infection risk may be clinic dependent. 10 (7 %) had attended only our adult CF clinic, 73 (51.7 %) had attended two clinics (generally the local pediatric CF clinic and the local adult clinic), 38 (26.9 %) had attended three, 13 had attended four (9 %) and seven had attended five or more clinics (5 %). There was no association with number of clinics attended and risk of ePA clinic 0/10 (0 %), two clinics 16/73 (21.9 %), and ≥3 clinics 14/58 (24.1 %), *p* = 0.30.

### Risk for acquisition of ePA

Risk factors were assessed for ePA by asking patients about exposures occurring in their childhood or early adulthood. Most notably, patients with ePA were more likely to have attended summer camps (Table 2). Furthermore, patients with ePA were more likely to have directly participated in CF fundraising activities which had social components associated with them although this did not extend to individuals with family members partaking in CF fundraising activities. Having a family member with CF did not increase risk of ePA infection.

**Table 2:** Presence of prior exposures/risks associated with risk of epidemic *P. aeruginosa* chronic infection

Exposure	Epidemic *P. aeruginosa* (*n* = 30) (%)	Compared to those with chronic unique *P. aeruginosa* strains			Compared to the rest of the clinic cohort irrespective of *P. aeruginosa* status		
		UNI (*n* = 66)(%)	OR	P value	ENT	OR (CI)	P Value
					(*n* = 112)		
					(%)		
Attended CF Camps	19/30 (63)	5/66 (8)	8.36 (3.45–20.3)	<0.001	8/104 (8)	8.23 (4–16.9)	*p* < 0.001
Personal Involvement in prior fundraising events	16/30 (53)	18/66 (27)	1.96 (1.17–3.28)	0.02	30/103 (29)	1.83 (1.16–2.87)	*p* = 0.02
Family member involved in CF fundraising events	16/30 (53)	25/65 (38)	1.38 (0.88–2.19)	0.128	37/102 (36)	1.47 (0.96–2.24)	*p* = 0.14
Have you ever lived with some one with CF	9/30 (30)	21/65 (32)	0.93 (0.48–1.78)	1	26/102 (25)	1.17 (0.62–2.23)	*p* = 0.64
Attended School or work with someone with CF	6/30 (20)	19/66 (29)	0.70 (0.31–1.56)	0.46	20/103 (19)	1.03 (0.46–2.33)	*p* = 1
Shared Medical Devices with someone with CF	6/30 (20)	10/66 (15)	1.32 (0.53–3.30)	0.57	12/103 (12)	1.47 (0.96–2.24)	*p* = 0.14
Shared Meals with someone with CF (outside of Camps)	10/30 (33)	17/66 (26)	1.29 (0.67–2.48)	0.47	21/103 (20)	1.17 (0.62–2.23)	*p* = 0.64
Intimate Contact with someone with CF^a^	3/30 (10)	5/66 (8)	1.32 (0.34–5.17)	0.7	4/103 (4)	1.03 (0.46–2.33)	*p* = 1

^a^Described as either kissing or a sexual relationship

### Knowledge regarding infection transmission potential

Patients with ePA did not significantly differ from those without regarding beliefs around risks and mechanisms of pathogen transmission (Table 3). Both groups recognized the risk of infection acquisition from aerosol, fomite and through health care workers (Table 3). In both groups patients demonstrated an understanding of pathogens with potential transmission potential and demonstrated a heightened concern regarding organisms such as *Burkholderia cenocepacia*, *P. aeruginosa*, and methicillin resistant *S. aureus* (MRSA) relative to other organisms such as *S. maltophilia*, and *Aspergillus* spp, and were not different amongst those infected with ePA (data not shown). Patients with ePA were more knowledgeable and concerned with respect to *B. cenocepacia* (Table 4). Both groups recognized that patients who have undergone transplantation were at increased risk of acquiring infections but participants were ambivalent as to whether those who had received transplant posed a risk for propagating infection.

**Table 3:** Beliefs regarding infection transmission potential^a^

Question	Epidemic *P. aeruginosa* (*n* = 30)	Compared to those with chronic unique *P. aeruginosa* strains		Compared to the rest of the clinic cohort irrespective of *P. aeruginosa* status	
		UNI (*n* = 66)	P Value	ENT (*n* = 112)	P value
Individuals with CF can spread germs to each other	6 (IQR 6–7)	7 (IQR 6–7)	0.54	7 (IQR 6–7)	0.87
*B cepacia* is a germ that can be spread	7 (IQR6-7)	6 (IQR 4–7)	0.04	6 (IQR 4–7)	0.03
*Pseudomonas* is a germ that can spread	6(6–7)	6 (6–7)	0.49	6 (6–7)	0.34
*Staphylococcus* is a germ that can spread	6 (6–7)	6 (5–7)	0.18	6 (5–7)	0.15
People with CF can spread infection via cough	7 (6–7)	6 (5–7)	0.14	6 (6–7)	0.2
Contaminated clinic surfaces such as chairs and desks can spread infection	6 (6–7)	6 (5–7)	0.57	6 (5–7)	0.36
Healthcare workers can spread infection	6 (6–7)	6 (5–7)	0.52	6 (5–7)	0.4
Patients with lung transplants are at risk of acquiring infections	7 (6–7)	6 (5–7)	0.3	6 (6–7)	0.26
Patients with lung transplants are at risk of spreading infection	5 (4–6)	6 (4–7)	0.97	6 (4–7)	0.65

^a^Strongly Disagree, 2. Disagree, 3. Neutral, 4. Neither agree or disagree, 5. Somewhat agree, 6. Agree, 7. Strongly Agree

**Table 4:** Patient beliefs regarding relative importance of an item in potentially propagating infection transmission^a^

Situation	Epidemic *P. aeruginosa* (*n* = 30)	Compared to those with chronic unique *P. aeruginosa* strains		Compared to the rest of the clinic cohort irrespective of *P. aeruginosa* status	
Situations associated with risk of transmitting infections		UNI (*n* = 66)	P Value	ENT (*n* = 112)	OR, P value
Patients not washing hands	6 (6–7)	6 (5–7)	0.69	6 (6–7)	0.84
Health care workers not washing hands	7 (6–7)	7 (6–7)	0.91	7 (6–7)	0.87
Clinic environment between patients	5 (4–7)	6 (5–7)	0.07	6 (4.5–7)	0.13
Exposure to another CF patient coughing	6 (6–7)	7 (5–7)	0.66	7 (5–7)	0.87
Exposure to another CF patient talking	4 (2–4)	5 (2–6)	0.1	4 (2–6)	0.16
Spirometry performed in a PFT laboratory	6 (4–7)	6 (4–7)	0.98	4 (4–6)	0.63
Importance of various measures in reducing risk of infection transmission					
Segregation of patients in to separate rooms	6 (5–7)	7 (5–7)	0.19	7 (6–7)	0.11
All patients washing hands/sterilizing with alcohol upon entry and exit from rooms	7 (7–7)	7 (6–7)	0.11	7 (6–7)	0.10
Patients wearing a mask in hospital common areas	4 (3–6)	5 (3–7)	0.44	5 (3–6)	0.98
Cleaning of examination rooms between patients	7 (6–7)	7 (5–7)	0.59	7 (5–7)	0.41
Avoiding *direct* physical contact with other patients	6 (3–7)	6 (5–7)	0.49	6 (5–7)	0.49
Avoiding *any* contact with other CF individuals	3 (2–4)	5 (2–7)	0.05	5 (2–7)	0.04

^a^No risk, 2. Very low risk, 3 low risk, 4 neutral, 5. Mild risk, 6. Moderate risk, 7. Extreme risk

direct = Any physical contact with an individual with CF

any = Any direct communication regardless of direct physical contact within the prescribed "2m" protective bubble

Whereas patients recognized the risk of infection transmission with failure to comply with mandatory hand hygiene on the part of staff and patients alike, both groups were less concerned about acquisition from the clinic environment (Table 4). Most notably, patients from both groups believed that infection acquisition was much more likely to occur during exposure to a CF patient coughing as opposed to merely talking. Furthermore, neither group was particularly concerned about the role of spirometry in aerosol generation.

We assessed several factors related to patient preference and attitudes. Patients were generally against grouping of individuals in common waiting rooms (ePA 3 (IQR 2–5) vs ENT 3.0 (IQR 1–4), *p* = 0.69), and preferred segregation in private rooms through their entire clinic visit. Patients generally suggested they were not concerned with acquiring infection in clinic; ePA 5 (IQR 2–6) vs ENT 5 (IQR 2–6), *p* = 0.93 and did not cite fear of infection as a reason for not attending clinic; ePA 1 (IQR 1–2) vs ENT 1 (IQR 1–2), *p* = 0.43. Few patients suggested they would prefer to be seen outside of CF clinic altogether, ePA 2 (IQR 1–4) vs ENT 2 (IQR 1–4), *p* = 0.64. However, socialization with others with CF was seen more commonly in those with ePA(Table 4). When asked if they would continue to socialize outside of CF clinic together patients with ePA were more likely to do so; ePA 5 (IQR 4–6) vs ENT 4 (IQR 2–5), *p* = 0.03 and UNI 4 (IQR2-6), *p* = 0.06. The same trend was observed in patients response to interacting on clinic days; ePA 5 (IQR 2–5) vs ENT 3(IQR 2–5), *p* = 0.05 and UNI 3 (IQR2-5), *p* = 0.08. Whereas past fundraising activities were observed to associate with ePA, participants did not agree that CF individuals should be excluded from future events; ePA 2 (IQR 1–4) vs non 2 (IQR 1–4), *p* = 0.5. Patients were ambivalent towards, and many reported being unaware of online social network platforms for those with CF; ePA 4 (IQR 2–5) vs non 4 (IQR2-5), *p* = 0.93.

## Discussion

ePA infection in CF, including infection with strains such as LES and PES, have been associated with increased risk of disease progression and worsened pre-transplant survival [2, 13]. Accordingly, strategies to prevent transmission of ePA are of paramount importance.

Risk factors for acquisition of ePA have often been difficult to ascertain owing to uncertainty regarding timing of infection in prospective studies. As our group has previously demonstrated that ePA acquisition occurs before or shortly after adulthood [7], we looked to identify which past behaviours were most closely associated with ePA. Herein, we identified the greatest risk factor for ePA acquisition to be during periods of prolonged close contact such as might occur during CF camps. Indeed these camps were run through many parts of the world, and several studies report instances of super-infection of 60–100 % of attendees [20, 21]. These camps were also associated with outbreaks of Bcc and have largely since been abandoned for this reason [22]. Whereas personal involvement in past social CF fundraising was observed to associated with ePA, current non-social fundraising activities either by patients or family members was not demonstrated to be a significant factor.

Others have reported significant gaps in infection control knowledge of CF patients and family members [14, 23]. In a multi-center survey involving 1399 patients, only 53 % of patients understood the risks associated with socialization and social behaviours such as hand shaking (57 %) or the rationale for the 1 m bubble (67 %) [24]. However, herein we observed, in general our population to be quite knowledgeable. We did, however, find that patients with ePA were less likely to believe in or adhere to clinic policy against socialization. Patients may believe that the benefit of a close personal connection to other individuals with CF outweigh the potential risk of infection transmission [14]. Others have reported that young adults are more likely to be non-adherent to infection control standards than other age groups, and this was postulated to be related to the fact these patients grew up spanning two very different periods of CF care. Indeed, these patients were also more likely to have attended camps where socialization was previously encouraged.

Droplet nuclei containing CF pathogens including *P. aeruginosa* have long been known to be created by those with CF [25, 26]. These have been shown to be the same strains causing chronic infection and to persist in the air for extended periods. However, in the absence of information regarding minimum infectious inoculum for ePA the risk of ePA in droplet nuclei within the clinic environment is unknown. In our study, patients associated coughing with heightened risk of infection acquisition but did not equate mere talking as a risk. Indeed, many health care works and patients believe that it is during periods of illness associated with increased coughing that the greatest risk of transmission occurs [24]. This is not the case as others have demonstrated that the amounts of infectious droplets generated during cough were similar during periods of stability and at exacerbation [27]. Indeed, *P. aeruginosa* in droplet aerosol are even identified during tidal breathing, albeit at lower levels then during cough. While use of masks has been advocated in the recent American infection control guidelines [28], recent work suggests that masks do not reduce risk of infectious aerosol generation [29]. This raises the potential concern of false security as use of masks may be viewed as a means amongst CF patients to facilitate safer socialization.

Strategies adopted for controlling the spread of ePA are quite diverse. Those advocated include: cohort segregation (where patients infected with ePA are seen at separate clinic/hospital facilities from those without) [30, 31], cohort segregation with environmental controls (whereby patients with any *P. aeruginosa* infection were seen separately in addition to strict environmental controls including faucet filters and splash back preventing sinks), and strict patient segregation incorporating the use of gowns and masks by patients and health care workers alike [32]. These increasingly complex strategies have all been attributed as being responsible for reduction in transmission. However, based on our observations herein we put forth that patient segregation, preventing socialization and the basics of hand and cough hygiene are the primary means of preventing ePA. Indeed, discussions about infection control knowledge and beliefs, and critically avoiding socialization with other unrelated CF individuals need to be addressed, and become part of the annual review [33].

Whereas prevalence of ePA are discouragingly high across multiple clinics, incident infections with these strains remain very low when simple measures are invoked [2, 7]. In prospective studies, in the few instances where ePA strains have been observed to superinfect patients, social connections have been identified [30, 34, 35]. Discouraging socialization thusly must be prioritized as ultimately the most important and controllable means of preventing ePA transmission. However, studies have suggested that CF care providers are often unfamiliar with the importance of this strategy, or may disagree entirely, and lack confidence in encouraging this practice [24, 36].

Within our cohort of patients we assessed if there was an association between number of clinics attended and risk of epidemic strains. While no association was found we did observe a high frequency of patients attending multiple CF clinics, including patients who had attended CF clinics in all regions of Canada, parts of the United States, Europe and Australia. As many surveillance projects rely exclusively on screening against local epidemic strains, we must remain mindful that focused strategies such as PCR targeting local epidemic strains may be insensitive to identification of novel or new strains as new strains may become established through patient transfers [30].

Interventions to improve infection control as it pertains to ePA must account for the fact that social connectedness appears to be the greatest risk factor. As this group seems also to be the most inclined to socialize and thereby potentially transmit infection, strategies both discouraging face-to-face contact while supporting the desire and perceived benefit of socialization need to be recognized. In this light the use of CF “online” social networking sites offer promise provided they can be adequately supported and regulated. Indeed, “offline” face-to-face meetings do occur and peer-to-peer or external safety measures must be continually emphasized. Whereas online message boards have traditionally been the means of communication, next generation peer-peer video chat and conference call systems are being developed and hold greater promise yet [37]. Unfortunately, uptake within the CF community remains limited and both promotion and continued expansion of this medium to meet the evolving needs of the community are required. Furthermore, social scientists can monitor these medium enabling great insight into the CF patient population with respect to beliefs, perception, concerns and understanding how patients individualize their disease and its management [38]. In an environment where health care resources are increasingly limited, and ever increasingly complex and expensive infection control protocols exist we suggest that investment in online platforms to enable these connections pose great “bang for the buck”.

## Conclusions

ePA infections are both common and associated with accelerated clinical decline. The most important risk factor for ePA infection remains peer-peer socialization, something now routinely discouraged. As patients continue to desire peer-peer support, novel strategies that enable this desire to be met are warranted. Investment in online peer-peer communication technologies represents a cost effective intervention for preventing further ePA transmission.

## Abbreviations

Bcc: *Burkholderia cepacia* complex
CF: Cystic fibrosis
CI: Confidence interval
ENT: The entire cohort who did not have ePA
ePA: Epidemic *P. aeruginosa*
IQR: Inter-quartile range
LES: Liverpool Epidemic Strain
MLST: Multi-locus sequence typing
MRSA: Methicillin-resistant *S. aureus*
MSSA: Methicillin-sensitive *S. aureus*
NTM: Non-tuberculous mycobacteria
PES: Prairie Epidemic Strain
PFGE: Pulse-field gel electrophoresis
UNI: Chronic stable unique *P. aeruginosa*
WGS: Whole genome sequencing
